# Supplementary material for: Relationship between demoralization of the college student with their individual- and social-oriented self
Source: PLoS One. 2022 Jul 6;17(7):e0271030. doi: 10.1371/journal.pone.0271030 (PMC9258854; doi:10.1371/journal.pone.0271030)
Supplement: Attachment — Submitted filename: PONE-D-21-20917 (Response to Reviewers).docx [file pone.0271030.s002.docx]

**PONE-D-21-20917: Relationship between Demoralization of the College Student with their Individual- and Social-Oriented Self**

**Review Comments to the Author**

Reviewer #1:

The manuscript is interesting with significant volume of informations. The research instruments are adequate for study organisation and qualitative data. The results and discussions should appear to be related with their individual social oriented self. The conclusions are relevant for the topic. I consider that the authors do a good job and the manuscript and the subject matter is very interesting for the journal.

**Reply:** Thank you very much for your positive remarks on this manuscript.

1. It seems that regression analysis is necessary to analyze the purpose of the study. Correlation analysis alone is problematic in interpreting the relationship between variables.

**Reply:** Thank you for your suggestion. We have taken the reviewer’s suggestion to treat demoralization scores as a dependent variable, and a multiple regression model was used to analyze the association between demoralization score and the measurement of individual-oriented self (ISS-I)/social-oriented self (ISS-S) among 356 participants. The findings of regression analysis were similar to correlation analysis. Demoralization overall score was correlated to increased competition, and decreased independence, self-determination, consistency and overall score in the ISS-I. Demoralization overall score was correlated to increased social sensitivity, and decreased contextual self, interpersonal relatedness and self-cultivation in the ISS-S. Therefore, we have modified the Table 4, please refer to *Page 14*.

**Table 4. Association between demoralization score and individual-oriented self (ISS-I)/social-oriented self (ISS-S) among 356 participants**

|  | Meaning of life | | Loneliness & helplessness | | Self-assurance | | Courage  and tenacity | | Emotional distress | | Demoralization  Overall score | |
| --- | --- | --- | --- | --- | --- | --- | --- | --- | --- | --- | --- | --- |
|  | *ꞵ* | *p* | *ꞵ* | *p* | *ꞵ* | *p* | *ꞵ* | *p* | *ꞵ* | *p* | *ꞵ* | *p* |
| ***Individual-oriented view of self*** | | | | | | | | | | | | |
| **Independence** | -.298 | <.001 | -.330 | <.001 | -.199 | <.001 | -.447 | <.001 | -.088 | .071 | -.334 | <.001 |
| **Self-determination** | -.129 | .015 | -.352 | <.001 | .009 | .874 | -.280 | <.001 | -.338 | <.001 | -.263 | <.001 |
| **Competition** | .111 | .026 | .152 | .001 | .326 | <.001 | .076 | .071 | .150 | .002 | .208 | <.001 |
| **Consistency** | -.255 | <.001 | -.190 | <.001 | -.206 | <.001 | -.193 | <.001 | -.302 | <.001 | -.284 | <.001 |
| *Overall score* | -.312 | <.001 | -.478 | <.001 | -.014 | .808 | -.495 | <.001 | -.469 | <.001 | -.426 | <.001 |
| ***Social-oriented view of self*** | | | | | | | | | | | | |
| **Contextual self** | -.169 | .002 | -.229 | <.001 | -.144 | .012 | -.300 | <.001 | -.001 | .984 | -.207 | <.001 |
| **Interpersonal relatedness** | -.264 | <.001 | -.086 | .136 | -.162 | .010 | -.006 | .919 | -.137 | .027 | -.168 | .004 |
| **Self-cultivation** | -.147 | .019 | -.309 | <.001 | -.039 | .549 | -.396 | <.001 | -.207 | .002 | -.263 | <.001 |
| **Social sensitivity** | .205 | <.001 | .310 | <.001 | .254 | <.001 | .222 | <.001 | .236 | <.001 | .306 | <.001 |
| *Overall score* | -.153 | .005 | -.036 | .489 | -.072 | .227 | -.139 | .005 | .095 | .079 | -.075 | .154 |

2. Previous studies are mainly Taiwanese journals. If you present a foreign journal together, it will be a better thesis.

**Reply:** Thank you for your suggestion. We have taken reviewer’s suggestion to add foreign journal of demoralization and modify the statement in the Introduction section on *Page 4-5.*

“Demoralization has been described as a psychological state characterized by helplessness, hopelessness, a sense of failure and the inability to cope (for a review, see Tecuta et al., 2015). Demoralization is defined as troubles in lives whereby affected individuals would feel helpless, meaningless, hopeless, remorse and so on when facing life problems [1, 2]. Clinically, demoralization is a new diagnostic concept when they discovered that patients with affective disorders and patients with schizophrenia showed the same psychological condition: loss of meaning, goals, and hope in life; a pessimistic attitude toward the future; alienation and lack of support; and lack of motivation to navigate through life [12]. Previous studies have purposed a various psychological well-being dimensions which are associated with an individual’s development of optimal functioning: positive evaluation of one’s self, the belief that life is purposeful and meaningful, the possession of quality relationships with others, a sense of continued growth and development, a sense of mastery over one’s environment, and a sense of self-determination [11, 13]. However, demoralization is opposed to psychological well-being. Previous studies have showed that patients demonstrated worse psychological well-being scores and significantly higher rates of demoralization [11, 14, 15].

Kissane et al. (2004) suggested that demoralization is a manifestation of existential distress, and they developed the first version of the Demoralization Scale, including five dimensions: such as loss of meaning, despair, disheartened feelings, helpless feelings, and a sense of failure. The scale was consisting of 24 items with 5-point Likert scale for participants’ self-report demoralization syndrome. In their study, they recruited 100 patients with advanced cancer (47 males and 53 females) to examine the internal consistency (Cronbach’s alpha coefficients ranged from .71 to .94) [16]. Moreover, factor analysis revealed five factors: loss of meaning (loss of meaning and worth connected to role and value in life), dysphoria (unspecific emotions of grief and regret), disheartenment (sense of isolation connected to frustration and loneliness), helplessness (subjective incompetence involving loss of control, hope and so on) and sense of failure (reversed sense of accomplishment, satisfaction and success in life translating into sense of failure).

Hung et al. (2010) translated Kissane’s Demoralization Scale into Mandarin version and tested its reliability and validity. They used the same five factors as Kissane et al. (2004), and the .928 Cronbach’s alpha was reliable. Hung (2019) modified the Mandarin version of the Demoralization Scale for investigating teenagers self-report demoralization syndrome, and five factors were meaning of life (satisfaction with the current life and self-identification), loneliness and helplessness (subjective feelings of isolation, lack of support or inability to receive assistance from others), self-assurance (sense of self-efficacy and understanding one’s own capability and strengths), bravery and perseverance (willingness to face difficulties and shoulder responsibilities), and emotional distress (feelings of regret, dysphoria, and lacking control).

Hung (2019) revised the scale to measure demoralization among high school students and to widen the application of the concept of demoralization. The demoralization experienced by high school students is similar to college students. Demoralized high school students and college students both experience subjective depression and agony, feel that no one understands them, and exhibit a certain level of suicidal ideation. However, some college students might show symptoms of depression such as loss of interest and social withdrawal. The differences between demoralization and depression inspired us to explore and gain a greater understanding of demoralization. Despite patients' demoralization has been well documented, knowledge about normal population and the related factors is still evolving. Hence, investigating college students offers a better understanding of their demoralization status and the cultural influences.”

We added the following references to the paper.

Kissane DW, Clarke DM, Street AF. Demoralization syndrome--a relevant psychiatric diagnosis for palliative care. J Palliat Care. 2001;17(1):12-21. Epub 2001/04/28. PubMed PMID: 11324179.

Ryff CD. Happiness is everything, or is it? Explorations on the meaning of psychological well-being. Journal of Personality and Social Psychology. 1989;57:1069–81.

Guidi J, Fava GA, Picardi A, Porcelli P, Bellomo A, Grandi S, et al. Subtyping depression in the medically ill by cluster analysis. J Affect Disord. 2011;132(3):383-8. Epub 2011/04/05. doi: 10.1016/j.jad.2011.03.004. PubMed PMID: 21458076.

Rafanelli C, Offidani E, Gostoli S, Roncuzzi R. Psychological correlates in patients with different levels of hypertension. Psychiatry Res. 2012;198(1):154-60. Epub 2012/03/06. doi: 10.1016/j.psychres.2011.09.014. PubMed PMID: 22386218.

3. The bibliography presentation in the text must be matched. Please match either year or number according to the academic form.

**Reply:** Thank you for your suggestions. We have taken the reviewer’s advice to recheck the bibliography presentation.

Reviewer #2: Background
1. Other than the meaning of life, the authors should also elaborate more on how "low individual- and low social-oriented self" would influence/inform other components of demoralization (e.g, self-affirmation, courage and tenacity, emotional distress).

**Reply:** Thank you for your suggestions. We have taken the reviewer’s advice to add the statements of unintegrated self in the Introduction. Please refer to *Page 8-9* for the changes made.

“Without proper guidance and sufficient self-exploration, college students may stay at unintegrated self with low individual- and low social-oriented views. They might lack motivation and enthusiasm for their work, even loss of meaning, goals, and hope. College graduates with unintegrated self entering the workforce are likely to experience contradictions and conflicts during the integration of two cultural values. To exacerbate matters, they may not know how to approach the situation, lose the meaning and goal of life, and eventually develop demoralization. Additional attention should be paid to this vulnerable group, and they should be provided with relevant support.”

Methods/Materials
2. The authors should mention how the total scores of demoralization, individual- and social-oriented self were computed.

**Reply:** Thank you for your suggestion. We clarified that the overall score of each scale was based on all dimensions; in addition, sub-scores were formed with 5-item in each dimension. Therefore, the overall score of demoralization was calculated the average of 25-item, including five dimensions; the overall score of ISS-I was calculated the average of 20-item, including four dimensions. We have taken the reviewer’s advice to add the statements to explain how the overall scores were computed in the “Data Analysis” of the Methods. Please refer to *Page 10* for the changes made.

“For each scale, the overall score was based on all dimensions; in addition, sub-scores were formed with 5-item in each dimension.”

We also modified the Table 3. Please refer to *Page 13* for the changes made

**Table 3. Demoralization overall score Among Four Groups**

| **Grouping** | **Demoralization overall score**  **(M ± SD)** | **F-value** | **Scheffe’s**  ***post-hoc* test** |
| --- | --- | --- | --- |
| Bicultural self (n = 99) | 2.26±0.50 | 18.44^***^ | U > B;  U > I;  S > B;  I > B |
| Individual-oriented self (n= 73) | 2.49±0.53 |  |  |
| Social-oriented self (n = 63) | 2.64±0.62 |  |  |
| Unintegrated self (n = 121) | 2.79±0.54 |  |  |

*Note*. N, number; M, mean; SD, standard deviation; B, Bicultural self; I: Individual-oriented self; S: Social-oriented self; U, Unintegrated self; ^***^*p* < .001

3. The authors should indicate if there are any previous studies supporting the validity of the two scales.

**Reply:** Thank you for your suggestion. We have taken the reviewer’s advice to add the statements and citations to supporting the validity of these two scales in the “Instruments” of the Methods. Please refer to *Page 10* for the changes made.

Mandarin version of Demoralization scale (DS-M-25)

“The Mandarin version of Demoralization scale (DS-M-25) was guided by the demoralization scale for cancer patients (Kissane et al., 2004) and its translated Mandarin version as DS-M-25 scale (Hung et al., 2010). In the DS-M-25 scale, the measurement (of demoralization) was including five factors comprising the following dimensions: life meaning; loneliness and helplessness; self-assurance; bravery and perseverance; and emotional distress. Five items were generated for each dimension, since the scale was designed for adolescents, negative-worded items were minimized to reduce the cognitive burden of participants. The 25 items using a 5-point Likert scale (1: *strongly disagree* to 5: *strongly agree*) to indicate the extent to which they agreed (or disagreed) with the items. Specifically, it shows good internal consistency and test–retest reliability for both adolescent and young adults (Hung, 2019). The instrument was similar to our prior study, which was including detailed reliability and validity of DS-M scale (Huang et al., 2021; Hung, 2019).”

Individual- and Social-oriented Self scale (ISS)

“The Individual- and Social-oriented Self scale (ISS) was developed with dual 4-factor structure of the ISS subscales encompassed the key components of the initial theoretical frameworks and broadly covers well-documented cultural differences in the self, including independence versus interdependence, cross-situational consistency versus context sensitivity, and other- versus self-focus (Lu, 2007; Lu, 2008). The 40-item ISS scale was a conglomeration of the individual- versus social-oriented self-dichotomy (Lu, 2008). The measurement of individual-oriented self (ISS-I) was including four factors comprising the following dimensions: independence; self-determination; competition; and consistency. In addition, the measurement of social-oriented self (ISS-S) was including four factors comprising the following dimensions: contextual self; interpersonal relatedness; self-cultivation; and social sensitivity. Five items were generated for each dimension using a 5-point Likert scale (1: *strongly disagree* to 5: *strongly agree*) to indicate the extent to which they agreed (or disagreed) with the items. According to previous studies, the ISS scale has revealed good reliability and validity (Lu, 2007; Lu, 2008; Lu et al., 2008; Lau, 2017; Chen, 2019).”

We added the following references to the paper.

Huang, C. Y., Hung, Y. C., Tan, C. S., Cheng, S. M. & Hu, S. H. (2021). Development and validation of a mandarin version of demoralization scale for adolescents and young adults. Current Psychology, https://doi.org/10.1007/s12144-021-02504-x.

Lu L. The individual-oriented and social-oriented Chinese bicultural self: testing the theory. J Soc Psychol. 2008;148(3):347-73. Epub 2008/07/09. doi: 10.3200/SOCP.148.3.347-374. PubMed PMID: 18605182.

Lu L, Kao S-F, Chang T-T, Wu H-P, Jin Z. The individual- and social-oriented Chinese bicultural self: A subcultural analysis contrasting Mainland Chinese and Taiwanese. Social Behavior and Personality An International Journal. 2008;36(3):337-46.

Lau Y-S. The Relationship among Family-Differentiation, Self-Efficacy, and Career Decision of College Student: Chinese Culture University; 2017.

Chen Y-T. The relationship among Taiwanese teenagers’ self-views, perceived parenting beliefs and learning motivations: National Sun Yat-sen University; 2019.

Data Analysis
4. Is there any reason(s) why the mean scores of the participants' individual and social orientation were used as the cutoff point? Given that the mean scores of "Independence" and "Situational self" were relatively high (over 4), low individual orientation/low social orientation groups may contain students who were high in independence and situational self.

**Reply:** Thank you for your suggestion. We have modified Table 2 with listing average of four groups (*Page 12*). Also, we clarified that the mean of the overall ISS-I/ISS-S score was used to divide into four groups, namely bicultural self (high ISS-I × high ISS-S), individual-oriented self (high ISS-I × low ISS-S), social-oriented self (low ISS-I × high ISS-S), and unintegrated self (low ISS-I × low ISS-S). We agree with your point. Despite the mean scores of Independence and Situational self were relatively high among four groups (Table 2), the overall ISS-I and ISS-S scores still were significant differences among four groups (*F*(3, 352) =110.34, *p* < 0.001; *F*(3, 352) =85.19, *p* < 0.001, respectively).

**Table 2. College Students’ Demoralization and Individual/Social-Oriented View of Self**

|  | Bicultural self  (n = 99) | Individual-oriented  (n = 73) | Social-oriented  (n = 63) | Unintegrated self  (n = 121) | All participants  (n = 356) |
| --- | --- | --- | --- | --- | --- |
| ***Demoralization*** |  |  |  |  |  |
| Meaning of life | 2.21±0.65 | 2.56±0.72 | 2.58±0.80 | 2.86±0.68 | 2.57±0.74 |
| Loneliness and helplessness | 2.51±0.77 | 2.67±0.77 | 2.46±0.86 | 2.58±0.77 | 2.46±0.77 |
| Self-affirmation | 2.07±0.62 | 2.31±0.74 | 2.60±0.82 | 2.79±0.72 | 2.56±0.79 |
| Courage and tenacity | 1.84±0.49 | 2.15±0.51 | 2.40±0.62 | 2.60±0.54 | 2.26±0.62 |
| Emotional distress | 2.65±0.63 | 2.76±0.64 | 3.14±0.69 | 3.12±0.64 | 2.92±0.68 |
| *Overall* *score* | 2.26±0.50 | 2.49±0.53 | 2.64±0.62 | 2.79±0.54 | 2.55±0.58 |
| ***Individual-oriented view of self*** | | | | | |
| Independence | 4.42±0.34 | 4.22±0.39 | 4.09±0.40 | 3.83±0.51 | 4.12±0.48 |
| Autonomy | 3.58±0.57 | 3.42±0.61 | 2.52±0.66 | 2.66±0.52 | 3.05±0.73 |
| Competition | 3.47±0.63 | 3.14±0.61 | 2.62±0.53 | 2.57±0.52 | 2.95±0.69 |
| Consistency | 3.44±0.71 | 3.18±0.63 | 2.45±0.70 | 2.48±0.56 | 2.88±0.78 |
| *Overall ISS-I score* | 3.72±0.36 | 3.49±0.21 | 2.92±0.20 | 2.88±0.23 | 3.25±0.46 |
| ***Social-oriented view of self*** | | | | | |
| Situational self | 4.32±0.38 | 3.88±0.43 | 4.27±0.39 | 3.82±0.48 | 4.05±0.48 |
| Interpersonal connection | 3.86±0.51 | 2.93±0.59 | 3.66±0.52 | 2.90±0.49 | 3.31±0.68 |
| Self-cultivation | 4.06±0.44 | 3.32±0.46 | 3.79±0.36 | 3.17±0.40 | 3.56±0.56 |
| Social sensitivity | 3.00±0.35 | 2.45±0.53 | 2.96±0.40 | 2.50±0.42 | 2.71±0.49 |
| *Overall ISS-S score* | 3.81±0.28 | 3.15±0.24 | 3.67±0.18 | 3.10±0.24 | 3.41±0.40 |

*Note*. M, mean; SD, standard deviation; ISS-I, individual-oriented self; ISS-S, social-oriented self

5. The authors should mention the statistical procedure used to rank the individual- and social-oriented cultural values.

**Reply:** Thank you for your suggestions. We have taken the reviewer’s advice to add the statements in the “Data Analysis” of the Methods. Please refer to *Page 10-11* for the changes made.

“Then, we used the mean of the overall ISS-I/ISS-S score as the cut-off point, and a 2×2 factorial design was allowed us to examine the effects of ISS-I/ISS-S (each with high/low levels) on their demoralization. Therefore, the participants were divided into four groups, namely bicultural self (high ISS-I × high ISS-S), individual-oriented self (high ISS-I × low ISS-S), social-oriented self (low ISS-I × high ISS-S), and unintegrated self (low ISS-I × low ISS-S).”

Results
6. Table 2 should report the descriptive statistics of the total scores of demoralization, individual- and social-oriented self. Similarly, table 4 should report the correlations between the total scores of demoralization, individual- and social-oriented self.

**Reply:** Thank you for your suggestions. We have added statements about the descriptive statistics of the total scores of demoralization, individual- and social-oriented self in Table 2 (*Page 12*). In addition, we added statements about the correlations between the total scores of demoralization, individual- and social-oriented self in Table 4 (*Page 14*).

**Table 2. College Students’ Demoralization and Individual/Social-Oriented View of Self**

|  | Bicultural self  (n = 99) | Individual-oriented  (n = 73) | Social-oriented  (n = 63) | Unintegrated self  (n = 121) | All participants  (n = 356) |
| --- | --- | --- | --- | --- | --- |
| ***Demoralization*** |  |  |  |  |  |
| Meaning of life | 2.21±0.65 | 2.56±0.72 | 2.58±0.80 | 2.86±0.68 | 2.57±0.74 |
| Loneliness and helplessness | 2.51±0.77 | 2.67±0.77 | 2.46±0.86 | 2.58±0.77 | 2.46±0.77 |
| Self-affirmation | 2.07±0.62 | 2.31±0.74 | 2.60±0.82 | 2.79±0.72 | 2.56±0.79 |
| Courage and tenacity | 1.84±0.49 | 2.15±0.51 | 2.40±0.62 | 2.60±0.54 | 2.26±0.62 |
| Emotional distress | 2.65±0.63 | 2.76±0.64 | 3.14±0.69 | 3.12±0.64 | 2.92±0.68 |
| *Overall* *score* | 2.26±0.50 | 2.49±0.53 | 2.64±0.62 | 2.79±0.54 | 2.55±0.58 |
| ***Individual-oriented view of self*** | | | | | |
| Independence | 4.42±0.34 | 4.22±0.39 | 4.09±0.40 | 3.83±0.51 | 4.12±0.48 |
| Autonomy | 3.58±0.57 | 3.42±0.61 | 2.52±0.66 | 2.66±0.52 | 3.05±0.73 |
| Competition | 3.47±0.63 | 3.14±0.61 | 2.62±0.53 | 2.57±0.52 | 2.95±0.69 |
| Consistency | 3.44±0.71 | 3.18±0.63 | 2.45±0.70 | 2.48±0.56 | 2.88±0.78 |
| *Overall ISS-I score* | 3.72±0.36 | 3.49±0.21 | 2.92±0.20 | 2.88±0.23 | 3.25±0.46 |
| ***Social-oriented view of self*** | | | | | |
| Situational self | 4.32±0.38 | 3.88±0.43 | 4.27±0.39 | 3.82±0.48 | 4.05±0.48 |
| Interpersonal connection | 3.86±0.51 | 2.93±0.59 | 3.66±0.52 | 2.90±0.49 | 3.31±0.68 |
| Self-cultivation | 4.06±0.44 | 3.32±0.46 | 3.79±0.36 | 3.17±0.40 | 3.56±0.56 |
| Social sensitivity | 3.00±0.35 | 2.45±0.53 | 2.96±0.40 | 2.50±0.42 | 2.71±0.49 |
| *Overall ISS-S score* | 3.81±0.28 | 3.15±0.24 | 3.67±0.18 | 3.10±0.24 | 3.41±0.40 |

*Note*. M, mean; SD, standard deviation; ISS-I, individual-oriented self; ISS-S, social-oriented self

**Table 4. Association between demoralization score and individual-oriented self (ISS-I)/social-oriented self (ISS-S) among 356 participants**

|  | Meaning of life | | Loneliness & helplessness | | Self-assurance | | Courage  and tenacity | | Emotional distress | | Demoralization  Overall score | |
| --- | --- | --- | --- | --- | --- | --- | --- | --- | --- | --- | --- | --- |
|  | *ꞵ* | *p* | *ꞵ* | *p* | *ꞵ* | *p* | *ꞵ* | *p* | *ꞵ* | *p* | *ꞵ* | *p* |
| ***Individual-oriented view of self*** | | | | | | | | | | | | |
| **Independence** | -.298 | <.001 | -.330 | <.001 | -.199 | <.001 | -.447 | <.001 | -.088 | .071 | -.334 | <.001 |
| **Self-determination** | -.129 | .015 | -.352 | <.001 | .009 | .874 | -.280 | <.001 | -.338 | <.001 | -.263 | <.001 |
| **Competition** | .111 | .026 | .152 | .001 | .326 | <.001 | .076 | .071 | .150 | .002 | .208 | <.001 |
| **Consistency** | -.255 | <.001 | -.190 | <.001 | -.206 | <.001 | -.193 | <.001 | -.302 | <.001 | -.284 | <.001 |
| *Overall score* | -.312 | <.001 | -.478 | <.001 | -.014 | .808 | -.495 | <.001 | -.469 | <.001 | -.426 | <.001 |
| ***Social-oriented view of self*** | | | | | | | | | | | | |
| **Contextual self** | -.169 | .002 | -.229 | <.001 | -.144 | .012 | -.300 | <.001 | -.001 | .984 | -.207 | <.001 |
| **Interpersonal relatedness** | -.264 | <.001 | -.086 | .136 | -.162 | .010 | -.006 | .919 | -.137 | .027 | -.168 | .004 |
| **Self-cultivation** | -.147 | .019 | -.309 | <.001 | -.039 | .549 | -.396 | <.001 | -.207 | .002 | -.263 | <.001 |
| **Social sensitivity** | .205 | <.001 | .310 | <.001 | .254 | <.001 | .222 | <.001 | .236 | <.001 | .306 | <.001 |
| *Overall score* | -.153 | .005 | -.036 | .489 | -.072 | .227 | -.139 | .005 | .095 | .079 | -.075 | .154 |

7. Given that the study hypothesized that "low individual- and low social-oriented self" would inform the development of demoralization, it is not clear that the findings of "competition" and "social sensitivity" contradicted the hypothesis.

**Reply:** Thank you for your suggestions. We clarified that the demoralization scores of the unintegrated group (low ISS-I × low ISS-S) was higher than the bicultural self (high ISS-I × high ISS-S) and individual-oriented groups (high ISS-I × low ISS-S), suggesting that unintegrated group might struggle in helplessness and hopelessness. However, Table 4 present the results among 356 participants. Therefore, we have taken the reviewer’s suggestion to examine the correlation between demoralization and competition of ISS-I/social sensitivity of ISS-S within the unintegrated group. For competition of ISS-I, there was a significant correlation between the competition of ISS-I and the loneliness & helplessness in the unintegrated group (*r* = .249), suggesting competitive students might feel loneliness and helplessness easily due to their unintegrated cultural value. No other correlations were significant in the unintegrated group. For social sensitivity of ISS-S, there were no significant correlations in the unintegrated group.

8. Were any variables considered as possible co-variates?
**Reply:** Thank you for your suggestions. We clarified that we have balanced variables such as sex and school region, so we didn’t treat any variables considered as covariates.

Discussion
9. A discussion of limitations of the study should be included.

**Reply:** Thank you for your suggestions. We have taken the reviewer’s advice to add the statements about the limitations of the study (*Page 18*).

“This study is limited by a lack of data to distinguish the developmental changes of the individual- and social-oriented views of self. Moreover, the small sample size of this study may not have enough power to demonstrate the influences of different cultural values. Therefore, it warrants further investigations with larger samples and different experimental designs to validate our findings.”
